# The Relationship between Ageism and Well-Being as Mediated through COVID-19-Related Experiences and Discourses

**DOI:** 10.3390/ijerph181910490

**Published:** 2021-10-06

**Authors:** Stefan Hopf, Kieran Walsh, Eilionóir Flynn, Nena Georgantzi

**Affiliations:** 1 Irish Centre for Social Gerontology, Institute for Lifecourse and Society, National University of Ireland Galway, H91 TK33 Galway, Ireland; kieran.walsh@nuigalway.ie (K.W.); nena.georgantzi@age-platform.eu (N.G.); 2 Centre for Disability Law and Policy, Institute for Lifecourse and Society, National University of Ireland Galway, H91 TK33 Galway, Ireland; eilionoir.flynn@nuigalway.ie; 3 AGE Platform Europe, 1150 Brussels, Belgium

**Keywords:** ageism, well-being, COVID-19, older persons, age identity, interpretation pattern

## Abstract

Both COVID-19 and ageism can have a negative impact on the well-being of older people. Yet, our knowledge on the links between COVID-19, ageism and well-being is still emerging. The present study aimed to contribute to this knowledge by exploring the lived experiences of older adults during the COVID-19 pandemic. To do so, we analyzed older persons’ subjective experiences and perceptions of ageism arising from COVID-19-related policies and discourses in two country contexts—Austria and Ireland—and the implications of these experiences for personal well-being. Based on the thematic analysis of 27 interviews with older adults, we found that participants perceived and encountered a discriminatory homogeneous representation of older people as a group. Three specific forms of this homogenization, namely stigmatization, paternalism, and scapegoating, were identified as impact on well-being. Moreover, our analysis showed how these forms of ageism challenge both the individual and social identities of older people, revealing older participants’ different attitudes in responding to this challenge. With reference to the international research literature, we discussed the impact of these experiences on the well-being of older people and the possible legal and socio-political implications of our findings.

## 1. Introduction

There are long-standing concerns and a growing body of research evidence that testifies to the negative impact of ageism-typically defined as age-based stereotypes, prejudice and discrimination [1,2]—on the health and well-being of older adults [3,4]. These concerns have been amplified, and perhaps most pronounced, during the Coronavirus 2019 (COVID-19) outbreak. With older people at a higher statistical risk of becoming ill or dying, [5,6], and susceptible to social isolation due to social distancing measures [7,8,9], the adverse effects of the disease on later life well-being were clear from an early stage [10,11]. However, so too was the potential for ageism and age discrimination (the behavioral component of ageism [12]) to construct, intensify and prolong these impacts. Civil society, e.g., [13] and research communities e.g., [14,15,16] were quick to warn of the proliferation of these patterns within pandemic related policies and discourse, and their damaging implications for older adults as drivers of social exclusion [17], loneliness [18], negative self-images [19,20] and poor well-being outcomes [4]. Such developments have a regressive effect on the efforts of the last three to four decades to replace the more negative biomedical and functionalist view of ageing with ideals and policies that promote an active, healthy and successful image of ageing [21].

Early policy manifestations of ageism during the pandemic included the application of chronological age limits to decisions concerning triage procedures in some settings [22] and the promotion of older adult specific isolation measures [23]. The accompanying public communication, where older people were homogenized as frail, vulnerable or helpless [14,24], pointed to an intensified form of ageism in the overall social discourse, and even a vilification of older populations in social media [25,26]. Despite concentrated efforts to gather representative insights on the impact of this period on older people, empirical evidence exploring the links between COVID-19, ageism and age discrimination, and well-being outcomes is understandably still emerging. There are also marked gaps concerning our knowledge of in-depth lived experiences, with cross-country comparisons of these experiences largely absent.

Relevant studies that are available primarily focus on the collection of survey data. For example, Nilsson et al. (2021), in a Swedish web survey of 1011 people aged 70–90 years showed how pandemic rhetoric and policies were experienced by older people as something that “turned them old” [27], with some respondents describing the categorization and homogenization (i.e., “old old” and at-risk groups) of the older population as ageism. Similarly, Reiner et al. [28] found that stereotyping, and offensive or hurtful content in the media and in policy was the most common form of ageism encountered amongst respondents. García-Soler et al. [29], in an online survey of 840 people aged between 18 and 84 years in Spain, found that ageist attitudes were widespread, with more than half of respondents indicating those over 65 years were a greater burden on the health and economic system and should be subject to more restrictions. This pattern was also evident in Ireland where according to Ward and Kenny 18% of Irish adults aged 60+ personally experienced negative attitudes or behavior on the basis of their age during the pandemic, with the majority of these experiences resulting from contact with younger people, and/or the utilization of health and retail services [30].

With regard to the impact of experienced ageism and age discrimination on personal well-being during COVID-19 the literature shows mixed results. While some studies reported only minor effects [27,28], other analyses highlighted negative consequences for the individual. Surveying the attitudes of 1092 people aged 50+ in Israel Cohn-Schwartz and Ayalon showed the impact of ageist (self-)perceptions and beliefs on personal well-being. According to their results, those older persons who believe that older adults are perceived as a burden to society reported greater dying anxiety and more age-based discrimination in relation to the pandemic [31]. Furthermore, there is evidence that pandemic related ageism is positively associated with stronger anxiety symptoms among older persons in general [32], and that those who experienced more ageism during the outbreak report poorer health and lower life satisfaction [33].

However, beyond these broad associations and relations, few studies have explored the lived experience of older adults in relation to ageism and well-being in the pandemic. Barth et al. [34] who conducted interviews with 20 older adults during the first wave of the COVID-19 outbreak in France identified both benevolent forms of ageism, such as patronizing behavior from family members, as well as negative manifestations like discriminatory age categorization. While benevolent forms of ageism resulted in feelings of infantilization and loss of autonomy, ageist stereotyping, and homogenization sparked outrage, especially because the imposed attribution of being ‘old’ did not fit with respondents’ self-perception. Wang et al., who completed 15 interviews with older Chinese immigrants in Canada found participants were concerned about “directives seeming to target ‘survival of the fittest’” and “that society seemed to be ‘giving up on seniors’” [35].

Furthermore, whether considering large-scale survey studies or small-scale pieces of research dedicated to capturing lived experiences, existing evidence overwhelmingly derives from single country contexts. We currently lack knowledge of how older people’s perceptions of ageism and discrimination, might differ across different legislative and policy contexts. Variations in approaches to ageism and age discrimination may influence the recognition of ageism, not to mention the assessment and articulation of ageism, and its links to COVID-19 and well-being.

## 2. Research Aim and Design

To address these gaps in knowledge, we explore older people’s subjective experiences and perceptions of ageism arising from COVID-19-related policies and discourses in two country contexts—Austria and Ireland—and the implications of these experiences for personal well-being. The data for this analysis comes from a larger project focused on older adults (60+) experiences with age discrimination in accessing services. All participants in the study therefore have personally experienced age discrimination in one of the following three areas: health, financial, or transport services. The timing of the data collection (between July 2020 and April 2021) facilitated an exploration of the impact of COVID-19 and the links between the pandemic and experiences of ageism or age discrimination.

The two countries were chosen due to their differences in national legislation on age discrimination and the political representation of older people’s interests, e.g., differences in relation to national age policies and the political and social standing of older adult interest groups.

With regard to legal protection against age discrimination, in Austria the scope of the national anti-discrimination law (the so-called Gleichbehandlungsgesetz (GlBG, BGBl I 16/2020)) is limited to the field of employment. In contrast, Irish equality legislation (Employment Equality Acts (EAA) 1998–2018 and The Equal Status Acts (ESA) 2000–2020) prohibits age discrimination in access to goods, services, housing, education, associations, in addition to employment. However, these provisions also include a number of exceptions where chronological age is not considered to be a discriminatory criterion, for example in relation to actuarial or statistical calculations. Despite these differences in scope, the definition of age discrimination is very similar in both jurisdictions, with discrimination involving an active “doing” in the form of an act, practice or rule that directly or indirectly disadvantages persons because of their age. This disadvantage is the factual component of discrimination, i.e., legally discrimination only exists in the case of less favorable treatment that causes a ‘real’ or objective disadvantage. In order to determine whether an act, decision or rule is less favorable, a comparator (a person/group who does not have the characteristic in question) and a comparable situation are also required in both settings (for Austria see: § 32 GlBG, BGBl I 16/2020; for Ireland see: ESA 2000–2020 Part I Section 3).

Furthermore, differences between these two countries exist in relation to the public health measures that were implemented in order to control the spread of COVID-19. In Austria, the first lockdown was imposed on 16 March 2020, followed by a second and third lockdown including night-time curfews between 17 November and 6 December 2020, and 26 December 2020 and February 2021, respectively. General protective measures comprised of an obligation to wear a face mask, social distancing regulations, contact restrictions, and bans on events and gatherings, with only a few exemptions, such as funerals. Except for the particularly rigorous isolation of nursing homes, there were no targeted measures for older people. Nevertheless, political attention and the media coverage focused on the particular risks for older adults, which is why this group was recommended to adopt a particularly high level of protective behavior. In Ireland, a phased locked down commenced from 12 March 2020 and by 27 March all state colleges, schools, crèches, non-essential services and businesses were closed, and a series of social distancing and social mitigation measures introduced. This included having to stay at home (unless to undertake essential travel/work), two kilometers restriction for exercise and travel and no gatherings with people from outside households. Cocooning guidelines for anyone over 70 years or medically vulnerable were also issued and encouraged people not to leave their homes. A second lockdown was imposed from October 21 to December 21, and a third lockdown was implemented from 30 December 2020 with a phased reopening from early May 2021. While travel and exercise restrictions varied somewhat over these periods, cocooning for those over 70 years has been encouraged up until the point of vaccination [36].

We begin by outlining the approach and methods employed in this research. This is followed by a presentation of the key findings of the analysis pertaining to COVID-19, ageism and well-being. The discussion positions these findings within the international literature and draws out the key contributions of the article in relation to ageism in later life, and approaches to ageing more generally.

## 3. Methodological Framework

We draw on the concept of ‘interpretation patterns’ to broadly frame our analysis [37,38]. In principle, interpretation patterns are a specific form of socially shared knowledge that is reproduced in the course of socialization and that structures our behavior, routines, attitudes, opinions, norms and values in regard to a particular topic [39]. As socially shared knowledge interpretation patterns are typically disseminated through (‘mass’) media [40]. Consequently, they underpin the societal representations of older age; in this case representations of older age, contextualized and situated amidst the COVID-19 pandemic.

In this article, we are in effect examining the subjective engagement of older people with these patterns against the background of the pandemic. Therefore, we focus on older persons’ opinions and descriptions of COVID-19-related policy measures and the representations of older age and older people in the public discourse. In order to explore how these perceptions and experiences impact older people’s well-being during the pandemic we draw upon the “eudaimonic” conception of well-being. In contrast to a hedonistic approach, which emphasizes subjective happiness and pleasure as central capacities of well-being, the eudaimonic perspective prioritizes self-realization in terms of autonomy, personal growth, self-acceptance, life purpose, environmental mastery, and positive relatedness [41,42]. Hence, we are interested in whether ageism or age discrimination experienced during the pandemic had a negative impact on older people’s perception and assessment of their autonomy or independence, and personal abilities or agency.

## 4. Materials and Methods

Data for this analysis comes from 27 in-depth interviews with 29 older adult participants across Austria and Ireland conducted by the corresponding author. The interview guide was based on a the problem-centered interview [43] and addressed four main topics: (i) personal attitudes towards ageing and older age; (ii) challenges older people are confronted with in general within each country; (iii) personal experiences of ageism and age discrimination in relation to service access including personal impacts, as well as coping and adaptive strategies; (iv) the impact of COVID-19 on everyday life and COVID-related experiences of ageism. While the analysis for this article concentrated on those parts of the interviews that are related to COVID-19, it is also informed by participants’ narratives and responses in relation to the other interview topics (e.g., interpretations of age discrimination as expressed in narratives about personal experiences of age discrimination).

Table 1 provides an overview of the participant sample, which includes two interviews in Austria that were conducted with couples. The 29 participants, comprising of 17 women and 12 men ranged in age from 64 to 86 years, with a median age of 72 years. Regarding socio-demographic characteristics, there are only very minor differences between the participants in both country samples. For example, all participants assessed their own financial situation as at least “fair” (never as “poor” or “very poor”). The Irish participants more frequently indicated a “good” or “very good” financial status (eleven persons in Ireland compared to seven in Austria). Participants were recruited through gatekeepers from national interest and advocacy organizations. Participant recruitment aimed for a sample size of 30 participants. Within this specifications participant sampling was based on the concept of theoretical sampling [44]. After an initial email exchange regarding information on the study, as well as the consent form, participants were contacted again by phone to arrange an interview date and to clarify any questions they might have. Data in Austria was collected in German via face to face interviews between July and November 2020, in between the first and second COVID-19 wave with most participants interviewed in their own homes. Some were interviewed in the offices of the gatekeeper organization. Irish interviews were conducted by telephone (in English), due to the COVID-19 situation in Ireland, the associated restrictions from October 2020 onwards, and the ethical and public health concerns in relation to conducting face-to-face interviews. Overall, the average interview lasted about 90 min. All interviews were audio-recorded and fully transcribed. Data collection was completed by April 2021. Ethical approval for the study was granted by the Research Ethics Committee of the National University of Ireland Galway. Informed consent was obtained from all individuals involved in the study.

Data analysis was based on a thematic approach as developed by Braun and colleagues [45] and carried out using the *Atlas.ti* software. The coding was first completed by one researcher. The results of this first step were then discussed with all authors. This process was repeated several times until there was agreement on the assigned labels. The coding process itself was characterized by a combination of deductive and inductive coding. First, a provisional coding framework related to COVID-19 was developed based on the key COVID-related themes addressed by questions within the interview guide. This framework was then refined through the coding process and expanded to incorporate inductive themes emerging from interview data collected during the discussion of non-COVID-19 specific topics. The resulting sets of codes were combined into thematic blocks, which represent the central themes described in the findings section.

## 5. Results

Before exploring the relationship between COVID-19, ageism and well-being, we begin with a description of the general well-being impacts of COVID-19 to contextualize the influence of the pandemic on participants’ lives.

### 5.1. COVID-19 and General Well-Being

The majority of participants reported coping relatively well during the pandemic and did not generally describe serious consequences for their own lives and well-being. Having access to a garden, a balcony or green spaces near their home, and becoming more involved with digital and online communication tools were two principal factors cited as limiting the negative impacts of COVID-19.

However, most participants also referred to restrictions on social distancing and mobility as a particular constraint that could impinge on feelings of social connectedness. When these measures prevented contact with family and children, the implications were more acutely felt resulting in feelings of despair and emotional distress in some cases. Furthermore, interviewees in both countries highlighted the ways in which ambiguities around COVID-19-related restrictions contributed to a sense of uncertainty around permitted forms and levels of engagement. Participants spoke about the lack of clarity and transparency with respect to the political communication of restrictions, which led some individuals to believe they were legally obliged to self-isolate, although this was not the case. It was clear in the accounts of a number of participants, that going outside for a walk was thought not to be permitted, as demonstrated in the quote from 72-year-old Patricia, who lives in a rural town in Ireland:

*“Now I broke the rules when I wasn’t supposed to break them, when I went walking. You know I was, I was out walking before I was supposed to be….”*

These misunderstandings effectively stripped an important coping mechanisms for some individuals.

For a small number of participants, pandemic related impacts on well-being were more severe, with these participants describing a range of negative outcomes. These experiences were generally intensified for Irish participants, where descriptions of feelings of anxiety were more frequent within participant accounts. In Anna’s case, it was the fear of coming into contact with other people, and possibly becoming infected, that played a decisive role in her withdrawal from social life during the pandemic. The personal distress that this caused was so severe, it led her to seek psychological help:

*“I’ve had ups and downs, personally yes, I have had ups and downs, I actually ah sought the help of a psychologist because my anxiety levels went up, I was very worried, I didn’t want to go out, I avoided going on buses and trains, I didn’t want to be near people, because of my age, naturally, this would be no other reason if I was honest”.*

*(Anna, 80, lives in a suburb in Ireland)*

For others, such as 74-year-old Joan it was the non-stop COVID-19-related media coverage and the constant reporting of death figures and age breakdowns that was especially stressful and frightening. Joan described how there was little escape from the attention given to pandemic, and thus the anxieties that it created:

*“[I tried] not to think about it. It’s just, you can’t not think about it because it’s just everywhere, everywhere and every night”.*

*(Joan, 74, lives in a rural town in Ireland)*

A number of other interviewees, particularly those who lived alone in rural parts of Ireland, like 64-year-old Frank described a feeling of “*cabin fever*”, and the sort of psychological strain that could stem from feelings of isolation and a lack of stimulation.

More generally within the interview sample, several participants spoke about the challenges to living a healthy lifestyle during the pandemic, and the consequent negative outcomes that could accumulate during the period. This included additional weight gain, and increased alcohol consumption.

### 5.2. COVID-19, Ageism and Well-Being

Ageism stemming from COVID-19-related policy and discourse emerged as a significant theme within the research. Although not the primary focus of the original study, COVID-19 was frequently referred to throughout many of the interviews and was harnessed by participants in both countries as an illustrative example of how they felt older people were regarded in the two societies. However, some respondents also regarded the measures and the efforts of the government as appropriate and necessary.

From an analysis of interview data, COVID-19-related ageism impacted on well-being in two ways, challenging first individual-level and group-level identity, and second, in a related manner, the positions of individuals and their older selves within society. Participants conveyed how ageism undermined fundamental attributes, around being active, being autonomous, and being in control, that were related to engagement and status within communities and societies.

First, findings in relation to the different forms of ageism that affected these attributes, and that reflect an external typification of ageing and older people during the pandemic, will be introduced. Second, findings will be presented on older people’s responses to these typifications, and the consequences for identity maintenance and re-construction broadly with respect to the eudaimonic concept of wellbeing, and especially in terms of older persons’ (self-perceived) autonomy and independence, personal capacities and individual agency.

### 5.3. Intertwined Forms of Ageism

Three primary interconnected forms or ageism were identified within the data as impinging on individual well-being. This included (i) stigmatization, (ii) paternalism and (iii) scapegoating. However, across all ageism forms, *homogenization* emerged as a base and integral component within their construction, with older people positioned as a uniform group during the pandemic. Here, homogenization refers to the discursive construction of a social group of people who in relation to a specific issue (the pandemic) share one or more supposedly relevant characteristics (e.g., chronological age) that is therefore socially perceived as a meaningful trait for grouping. As each form of ageism then builds upon this assumption to construct their own particular challenges for older participants, they effectively represent different sub-categories of homogenization. Before discussing the three subcategories, illustrations of homogenization will be first presented.

#### 5.3.1. Homogenization

The central basis of COVID-19-related homogenization to emerge from the data was chronological age. For many participants, such as Maria, the grouping of “everyone over 65” neglects the differences between the life circumstances of different age cohorts, and the diversity of different sections of ageing populations. Furthermore, this externally ascribed group membership is perceived as a denial of one’s own personhood (“you’re not Ms. Maria”), and individual differences in relation to, for example health and mobility:

*“You just belong, you’re just the risk group, you’re not Ms. [Maria] or whatever, you’re just the risk group (…) that’s it, your state of health doesn’t really count anymore, […] it is an imposed label for everyone over 65, whether they are fit or not, whether they are mobile or not, they are all in the same group, […] because those who are 80 are at risk group, those who are 90 and those who are 100, […] from this point on you belong to it”*

*(Maria, 67, lives in a suburb in Austria)*

A similar description is offered by Aileen in relation the Irish “cocooning-policy”. In this quote, not only is the indiscriminate grouping of older people into one category critically emphasized, but also the characteristic of chronological age used for this purpose.

*“Old people were put into that category of over-70s and that we were to cocoon, and we were not to go out and […] to stay home, […] all because we were over 70, and that I found very ageist”*

*(Aileen, 74, lives in a suburb in Ireland)*

In addition to illustrating the homogeneous grouping of people on the basis of age, these two quotations point beyond the basic problem of homogenizing older age to its link with the subcategories. Maria highlights the stigmatizing nature of older people as the “risk group”, while Aileen draws attention to pandemic-related paternalism (“because we are over 70”).

#### 5.3.2. Stigmatization

The stigmatizing form of ageism was primarily evident within participants’ descriptions of their unease in relation to the use and proliferation of the “risk group” label and its related associations. While many of the participants acknowledged the greater likelihood of poor COVID-19 outcomes with advanced age, they expressed concern, just as Niamh does, that all older people were being deemed as vulnerable and frail.

*“Well, older people were portrayed as more vulnerable, more at risk. I think we were definitely portrayed as the weakest. And again, I’m not sure how true that is […] I think again it’s a blanket portrayal. I think that lots of older people were less vulnerable”*

*(Niamh, 68, lives in a city in Ireland)*

Niamh’s quote again illustrates the link between stigmatization and homogenization. For her, the “blanket portrayal” of older people in public discourse is in clear contradiction with the actual situation of many older people; a group to which she counts herself (“we”) as a member.

For other interviewees this change in the portrayal of older persons marked a regression in public and political language. The transformed and promoted image of older age as a healthy and independent phase of life had seemingly been abandoned during the pandemic, with the notion of the ‘active’ older person being replaced by that of the ’fragile’ older person.

*“The image of older people was turned around 180 degree, from those who like to consume, who like to go out for dinner and travel, and stay fit […] and now, all of a sudden, they are completely fragile and at risk and are people who have to be cared for, and who are also somehow a burden to us, because we have to provide so much care, so much help for them and have to protect them so much […] and one believes one is allowed to take responsibility for them“.*

*(Marta, 62, lives in a city in Austria)*

As apparent in Marta’s quote, interviewees conveyed two key attributes with respect to this regression. First, participant’s described dismay in the ways that older people were constructed as a burden to society that threatened the capacity of a society and its systems to respond to the crisis. Second, is the perception that society has to care for older persons and assume protection for their well-being. This points to the second thematic subcategory, that of paternalism.

#### 5.3.3. Paternalism

The paternalizing treatment of older people was both addressed as a general issue by participants, and a by-product of specific pandemic-related measures. Closely related to aspects of homogenization and stigmatization, paternalistic forms of ageism were exemplified by political decisions, which aimed to reduce risk but also restricted freedoms, without the consultation of the older population. As Cornelia describes for many participants this represented a direct form of exclusion that compounded having to remain at home:

*“What was most difficult for the old people during COVID […] was that we were not asked whether we wanted to be locked up […] and I was at home and I was boiling inside and I said that’s discriminatory, [the older people] stay at home and the young can go out”*

*(Cornelia 76, lives in a city in Austria)*

Seventy-four-year-old Aileen from Ireland offers a similar description, highlighting an almost strategic marginalization under the auspices of the provision of safety. Aileen “*felt quite angry about the cocooning*” which she thought was “*very ageist*”, because although it was meant as a protection “[…] *it felt* […] *that you were being sort of pushed away or pushed out of sight and into supposed safety*”. As expressed in these and earlier quotations, participants highlighted the patronizing nature with which these measures were framed, and their exclusionary outcomes with respect to social participation and self-determination.

A more specific example of perceived paternalism that we repeatably found in our data related to the supermarket time slots allocated to older people. For interviewees, while the time slots may have been based on good intentions, they conveyed a feeling of paternalism. For Stefan, it remains unclear as to why he should only go shopping at certain times, since he adheres to all safety precautions:

*“Yes, they told us that, and then you start thinking, why did they say that people over 75 should go shopping between 9 and 10 o’clock, I don’t have anything and I’m wearing a mask, I go there, wash my hands, use disinfectant but why did they keep saying that I shouldn’t […] so there is this paternalism, you get the feeling”.*

*(Stefan, 78, lives in a rural town in Austria)*

Several other interviewees also stated that they had not made use of these allotted time slots and had instead completed their groceries at a time that suited them best. These participants also emphasized that they were capable themselves to assess when grocery shopping would be safe (e.g., fewer people; smaller queues), and did not require these prescriptive considerations.

#### 5.3.4. Scapegoating

The category of ‘scapegoating’ related to the perception of respondents’ that older people were being blamed for, what Erwin (62) calls, the “locking down” of society.

*“…no one wrote that the older people are to blame, but in fact, by saying again and again that I have to take these and these measures so that I can protect the older people, no one is explicitly blamed, but the result is the same”.*

*(Erwin, 62, lives in rural town in Austria)*

Underpinning the attribution of this blame is again the homogenization and stigmatization of the older population as a risk group that needs to be protected. John explicitly describes the mechanisms at work here. Older people are blamed for the effects of the pandemic based on the assumption that all older people are labelled as “sick and ill”.

*“I can say about the COVID-19, […], pick a scapegoat and the scapegoat is anyone over 65 or 70 or whatever it is, what they don’t realise is that […] not all old people are sick and ill and some old people are very well. […]. They’re turning old people into a scapegoat”.*

*(John, 75, lives in a city in Ireland)*

These quotes illustrate that the older people we interviewed have indeed been aware of the ageist discourse and the ageist portrayal of older people during the COVID-19 crisis, and that they critically reflect on it.

### 5.4. Age-Identity Construction in Times of COIVD-19

In addition to accounts of these three different forms of ageism, interviewees also addressed, implicitly and explicitly, their reaction to this ageism. These descriptions demonstrate the impact of the pandemic on the construction of subjective age-related identity in response to the external ageist typification. Data analysis identified three ‘*attitudes*’ as to how interviewees react to this external attribution: “the resistant”—those participants who resisted these typifications; “the compliant”—those who complied with the external attributions; and “the ambivalent”—those who express an ambivalent attitude.

#### 5.4.1. The Resistant

The first group of participants is characterized by their rejection of the external attribution. Their own age identity is then constructed in contrast to the discursively conveyed image of older age that participants perceived to have emerged during the pandemic. This can happen offensively, in that participants explicitly “resist”, as the following quote from John shows, who is talking about the measures implemented for ageing populations in Ireland:

*“What they [politicians] don’t realise is that people who are in nursing homes are generally speaking they’re old people because, but all old people are not in nursing homes and all old people are not sick and ill, and some old people are very well. I’m very well and I don’t have the virus and I know I’m very happy I’m not going to die of the virus”.*

*(John, 75, lives in a city in Ireland)*

John clearly rejects the image of older age as a “sick and ill” phase of life. In contrast, he is “*very well* […] *and not going to die from the virus*”. This narrative is consistent with John’s account of his experience of age discrimination in the context of financial services. According to John’s description he would not get a loan because “*statistically* […] *the average person of* [his] *age probably only has a couple of years left*”, but he himself is an “*outlier on the statistics*”. Central to the resistance that pervades these descriptions is their basis in their own right on an implicit ageism. The construction of John’s age identity is based on the demarcation from ‘old people’ in nursing homes, as those older adults who, in his opinion are actually at risk.

Other participants did not appear to explicitly oppose the externally imposed attribution, but instead describe how it did not simply fit with their self-perception. This construction reflects the second form of the resistance attitude.

*“I mean, on the other side, on the other side there was the, well, risk group, so there is then one’s own age again. So that numerical age [I] became aware [of], and I realised again that I don’t feel like that”.*

*(Hilde, 65, lives in a rural town in Austria)*

Thus, the attribution of being part of the risk group due to her own age has reminded Hilde of her chronological age, which she cannot identify with. This attitude is also based, albeit less explicitly than in John’s case, on certain normative expectations about how one feels at a certain age. The reference to the pandemic and belonging to the risk group thus illustrates that these are negatively connoted expectations.

#### 5.4.2. The Compliant

This second attitude reflects those participants that accepted or saw some congruence between the externally conveyed images of ageing and older people, and their subjective notions of self and identity. In these cases, a previously positive view of one’s own chronological age is devalued by the characteristics attributed to this chronological age during the pandemic, as Maria illustrates.

*“I personally felt older than I was up to that point. Well, up to that point I thought, well, I do this and this and this, I make sure that I don’t break anything [any limbs], but that I’m already so old (laughs). Somehow I’ve become more aware of that again…”.*

*(Maria, 67, lives in a suburb in Austria)*

For Maria, the image of older persons that emerged during the pandemic contributed to feeling older than she “*was up to that point*”. As a consequence, she readjusts her own view of and expectations in relation to her chronological age. Therefore, while Maria had previously only taken trivial ‘precautions’ (“*I make sure I don’t break anything*”), she has now become aware of being “*already so old*”. In the context of the pandemic, this means being at risk, being vulnerable and being in need of protection. Hence, in Maria’s case, her subjective age identity has changed in a rather unfavorable way.

In contrast to Maria, the following quote from Kaleb illustrates not an adaptation but a correspondence between the externally ascribed and subjectively constructed age identity.

*“Yeah, no, well my thoughts on all of that in the COVID environment is I’m at an age where I have to be very careful, I have a few health issues; I have to be very careful, that I can ill afford to get COVID. Whereas if I was twenty or thirty years of age, I could get COVID and I would weather the storm, I’d be fit, I’d be capable, […] but the people that are dying are my age group. So I’ve just been very careful, that’s what I am, I’m just careful about it, and that’s when you… there’s a vulnerability there, you know”.*

*(Kaleb, 70, lives in a suburb in Ireland)*

For Kaleb, his age places him in the risk group. Although he also mentions some health constraints, the comparison with other more resilient age groups (“*if I was twenty or thirty years of age* […] *I would weather the storm*,”) as well as his description that the people who died from COVID-19 were from his age group emphasizes age as a risk factor. This is further stressed by his emphasis on vulnerability and its association with his age group.

#### 5.4.3. The Ambivalent

The last attitude can be characterized by a certain degree of ambivalence. The age identity attributions are not completely rejected, but nor are they accepted uncritically, or without negative emotions. Thus, the narratives are not directly about the rejection or appropriation of socially prescribed age identities, but about the context that prioritizes age as a relevant distinguishing characteristic.

*“… it really bothered me […] they said subliminally to a certain group of the population that we are shutting everything down just because of you, […] that was the first time I became a bit aware of my age […] this general, so to speak, yes, the older people are so endangered and therefore we have to […] shut down, […] but this was the first time that I became a somewhat aware of older age, so to speak. Am I now with the group of people who have to be protected?”.*

*(Erwin, 62, lives in a rural town in Austria)*

However, for participants in this research, and reflecting the original focus of the study on difficulties accessing services, experiences and perceptions in relation to ageism and COVID-19 were sometimes contextualized within personal experiences and broader concerns about the treatment of older people in Austria and Ireland. As an example, in the following quotation Maureen talks about her experiences of discrimination in relation to having to pay increasing travel insurance premiums on the basis of her age, and about a train journey where she felt discriminated against by a conductor’s language and attitude. This helps to illustrate the societal devaluation of older age as a pre-existing pattern of ageism that is being perpetuated and possibly intensified during the pandemic:

*“Well, regards to the train, it certainly upset me because it made me feel I’m growing old and it made me feel that I was being treated like a piece of dirt, that I was in the way. In regard to insurance, it certainly makes you a lot more aware that you’re getting older, that you’re being discriminated against and it makes you feel as if you’re no longer a citizen of the world”.*

*(Maureen, 76, lives in a rural town in Ireland)*

Taken together, the two quotations within this section exemplify how situations in which older people become aware of their age can have negative connotations, in this case discrimination. In other words, in older age, those situations in which age comes into play as a significant category of distinction, i.e., becomes relevant as a reference category that evokes certain expectations, actions, regulations or language seem to be negative events.

## 6. Discussion

The aim of this article was to explore older people’s subjective experiences and perceptions of ageism arising from COVID-19-related policies and discourses in Austria and Ireland, and the implications of these experiences for personal well-being. As such, this analysis attempts to respond to the key gaps in knowledge around experiential insights in relation to how older people perceive, encounter and react to ageism in the context of COVID-19, and in relation to the absence of cross-country comparisons. Without addressing these deficits, we will lack an in-depth understanding of the mechanisms of ageism that can arise during such macro-level societal shocks to challenge individual well-being, and the influence of differential cultural and legislative contexts in shaping these mechanisms, and our response to their impacts.

From the outset it is important to acknowledge a number of limitations to this analysis. The design of the data collection was opportunistic and as a result the focus that could be given to COVID-19 specific circumstances was more constrained than what it might have been in a dedicated study. Notwithstanding our interest in capturing experiences in depth, the number of participants within this research limits the diversity of views that can be represented within the analysis. In a similar respect, while there was a reasonable balance in the number of participants across gender and socio-economic groupings, the sample does not adequately reflect the circumstances of people belonging to other social groups, based for example on ethnicity, sexual orientation or disability, or to traditionally hard to reach older populations (e.g., urban deprived, and remote rural residents). This limits our capacity to elaborate on the meaning of the findings for intersectional identities, and marginalized groups who are likely to experience exacerbated forms of disadvantage during the pandemic c.f. [35]. Another limitation concerns the age distribution, as the study sample does not include people who are 90 years old or older. It must also be recognized that the scale of the study, and the scope of the analysis presented in this article, means the influence of cultural context is difficult to fully disentangle and deserves a more involved investigation in future cross-country comparisons on this topic. Finally, as this research included participants who had already experienced some form of ageism or age discrimination in accessing services, it might be argued that interviewees were more likely to interpret circumstances through this position of perceived disadvantage/inequality. We acknowledge that this is a bias that may be operational within people’s accounts. However, we also recognize that such a unique positionality may provide this group with a reference-base to aid their assessment of COVID-19-related policy and discourse. This may also mean they are more attune to ageist mechanisms—an attribute that is likely to be valuable given that older people have sometimes been noted not to identify their experiences as a product of age-related discrimination [46,47].

We now turn to the findings of our analysis and the explicit contributions of this article. The research indicates that while the links between COVID-19 in general and social and psychological aspects of well-being are not clear-cut, the pandemic, and the related public health measures had significant consequences for the welfare of a number of participants in this study. The research also demonstrated the capacity and agency of older people to harness resources and adaptive strategies to ameliorate and to adapt to adverse circumstances within their daily lives. However, this has been a limited exploration within our analysis, and the direct links between COVID-19 and well-being are usefully investigated in more detail within other articles in this Special Issue.

From an analysis of interview data, COVID-19-related ageism impacted on wellbeing in two ways, challenging first individual-level and group-level identity, and second, in a related manner, the positions of individuals and their older selves within society. With regard to participants’ subjective perceptions of the pervasiveness of ageism during the COVID-19 pandemic, our findings are not unexpected and support the empirical results of similar studies within the international literature [28,29,34]. However, the main contribution of this article has been to draw out how the external interpretation patterns of older age which are evident within COVID-19 pandemic related policies and discourse, are interpreted by older people. While these patterns reflect the characteristics and nature of ageism that have been highlighted by civil society and academic communities during the pandemic, this article has demonstrated how older people understand their operation within society, and by in large their meaning for the social positioning of ageing and older people. Implicit within many of the quotations and narratives is interviewees’ recognition of the exclusion or denial that is associated with such patterns. Regardless of form, or any good-natured sentiment of protection that may underpin certain measures (e.g., dedicated supermarket slots), it was clear that these ageist categories were considered to undermine individual identity and also convey, and thereby re-produce, a strongly devalued group identity. These external projections reconstitute lived realities within a discursive frame of what it means to be old in times of a pandemic, where older people are defined by their chronological age, are vulnerable and frail, and are not capable of independent self-care, or indeed are to blame for the consequences of the crisis. Thus, what could be argued to be the more traditional negative interpretation patterns of older age have been reinvigorated by the pandemic to challenge older adults’ self-images; a finding echoed in comparable studies on COVID-19 [27]. Based on the well-researched relationship between older adults’ self-images and their well-being [19,20,48,49,50], we can conclude that as these interpretation patterns influence older adults’ self-images, they thereby impact on their well-being, In our analysis this relationship was borne out by the resistant, compliant and ambiguous attitudes evident in participant descriptions of how they responded and reacted to the prevailing ageist patterns. These findings can be linked to existing insights regarding the construction and appropriation of age identities and the influence of ageism on this process. The rejection of the attribution of “being old”, which corresponds with the attitude of “the resistant “, is empirically well documented. Older people not only reject the label “old” [51] but typically report feeling younger than their chronological age and express a certain degree of “agelessness” [52,53,54]. Equally well researched is the attitude of some older people to regard themselves as “being too old” to perform certain activities or tasks [55]. Such perceptions are socially mediated, i.e., they are acquired in the course of socialization [56] and maintained or reinforced by corresponding social narratives and can then manifest themselves in the form of “self-ageism” in older age [19]. If such negative social images of older age are adopted in the self-image, this requires a corresponding “compliance”.

This finding raises questions about the opportunities for creating and maintaining a (positive) subjective (age) identity that are offered to older people by modern societies, and thus which social interpretations of ‘older age’ are being embraced within those societies. It also raises concerns about the validity of our progress in transforming and re-framing later life as a multifaceted experience of engagement and participation. While promoted as breaking from the narrow, homogenizing view of ageing being dominated by health concerns and biological decline [21], concepts such as active, healthy, positive and successful ageing have, as suggested by Marta, fallen away since the onset of the pandemic. It is here where perhaps the critiques of these concepts, in themselves suggested to be homogenizing, come to the fore. Such positive affirming constructs and their related policy goals have long been challenged for neglecting the positions of heterogenous populations and marginalized groups, with questions raised about what active and healthy ageing might actually mean for people who may have less resources in social, economic and health domains [57,58]. Advancements in these various different agendas have certainly been made over the years. However, our failure at policy and societal levels to fully interrogate these ideas for diverse and potentially more vulnerable sections of the older population c.f. [59,60] is likely to partly explain their disappearance from ageing-related discourse during the pandemic, when their applications are confronted by circumstances that accentuates the vulnerabilities and risks of some of these population sub-groups. It might be argued that COVID-19 has as such exposed the shallowness of such concepts, and their lack of capacity to truly underpin the development of equitable ageing societies. It is possible to conclude from this perspective that “doing age” [61] in older age continues to be embedded in negative social practice. In addition, while these typifications suggest the operation of forms of internalized ageism, we could not explore this in detail within the scope of our analysis. Due consideration should be given to examining the implications of internalized representations, and particularly in relation to the long-term impact on these cohorts of older adults.

Finally, another strength of this study is the possibility to compare the experiences of older people from two EU countries. For our analysis, the differences in the legal protection against age discrimination, in the political institutionalization of the interests of older people and in the measures to mitigate COVID-19 were the most important to consider. The more comprehensive legal protection, as it exists in Ireland, could for example contribute to an increased legal awareness of age discrimination [62]. Similarly, Ireland’s comparatively stricter COVID-19 policy would seem predestined to allegations of ageism, as elements of these measures were based on chronological age. Yet, we do not find major differences between the countries in our participants’ comments on perceived ageism—except where some Austrian interviewees take a more critical view. This lack of major differences may be due to the fact that the public discourse on the individual and societal dangers of COVID-19—at least in Europe—focused almost exclusively on older people and those with pre-existing health issues. The lack of differences may thus be interpreted as an indication of the uniformity of the stereotyping and homogenizing discourse about older people across language, legal and cultural boundaries. This also raises the question whether current approaches to combating ageism and age discrimination, such as more extensive legal protection, are sufficient to raise awareness of ageism and age discrimination as social injustice. Although some participants from both countries voice critical perspectives, these are rarely embedded in any kind of overarching justice or equality discourses.

## 7. Conclusions

What qualifies as age discrimination and what can be considered as justified differential treatment is difficult to disentangle. After all, ageism and age discrimination are so pervasive and insidious that they may go unnoticed or remain hidden behind a range of explanations and justifications, which are perceived as neutral or necessary despite imposing an undue burden on the older population (Law Commission of Ontario. https://www.lco-cdo.org/en/our-current-projects/a-framework-for-the-law-as-it-affects-older-adults/older-adults-funded-papers/ageism-and-the-law-emerging-concepts-and-practices-in-housing-and-health/ii-ageism-concepts-and-theories/ (accessed on 21 July 2021)). This is the case for many legal regulations, policies and practices which emerged during the COVID-19 pandemic, including the imposition of blanket cocooning or shielding requirements on people over a certain chronological age, regardless of their personal health status [63]. The present article addressed this kind of ageism and age-based discrimination, which, because it is benevolent (e.g., supermarket time slots) or statistically underpinned (risk grouping based on chronological age), may not be perceived as discriminatory or can be justified legally because it is presumed necessary and proportionate in pursuit of a legitimate aim, i.e., to protect public health or safety [64]. Yet, the failure to recognize practices, like those represented within homogenization, stigmatization, paternalism and scapegoating within pandemic related legal regulations, policies and public discourse may contribute to the perpetuation of systemic ageism. Consequently, the adverse impact of age-based measures and the ageist discourse in the context of COVID-19 on older people’s well-being demonstrates the need for further scrutiny in the criteria used to design and justify such measures.

What does this imply in terms of future (crisis) policy? First and foremost, policymakers and legislators need to become aware of the interpretation patterns of older age that underlie and that are reproduced by their policies and their rhetoric. Secondly, we need a better understanding of the impact of these interpretation patterns and the typifications they encompass on the self- and societal images of older age and older people. This, as we have shown, is necessary because these typifications can impact on the well-being of older people. Consequently, social scientists and policymakers need to think about viable options to avoid a relapse into supposedly outdated social interpretations of older age. In the long run, the main challenge will be to scrutinize the potential of current ageing policies, which promote images of healthy, active, productive and successful ageing, to permanently shape the social perceptions and interpretations of older age. Perhaps the interpretation patterns contained in these positive ageing policies prove to be too homogeneous, too one-dimensional and too idealistic to provide the necessary social conditions and framing that could enable the increasingly diverse older population to have equal opportunities to develop a positive age identity.

## Figures and Tables

**Table 1:** Overview participant sample.

Participant Nr.	Country	Name (Pseudonym)	Age	Residence	Date of Interview
1	Austria	Marta	62	City	09/07/2020
2	Austria	Helga	80	City	15/07/2020
3	Austria	Cornelia	67	City	16/07/2020
4	Austria	Friedrich	67	City	21/07/2020
5	Austria	Irene	78	City	21/07/2020
* 6	Austria	Stefan	78	Rural Town	30/07/2020
* 7	Austria	Romy	80	Rural Town	30/07/2020
8	Austria	Konrad	86	Rural Town	30/07/2020
9	Austria	Fritz	78	City-Suburb	06/08/2020
* 10	Austria	Erwin	62	Rural Town	20/08/2020
* 11	Austria	Ingrid	64	Rural Town	20/08/2020
12	Austria	Maria	67	City-Suburb	25/08/2020
13	Austria	Gregor	71	City	26/08/2020
14	Austria	Roland	77	Rural Town	15/09/2020
15	Austria	Hilde	65	Rural Town	14/10/2020
16	Ireland	Anna	80	City-suburb	22/12/2020
17	Ireland	Martha	64	City	19/01/2021
18	Ireland	Niamh	68	City	22/01/2021
19	Ireland	Joan	74	Rural Town	29/01/2021
20	Ireland	John	75	City	01/02/2021
21	Ireland	Patricia	72	Rural Town	05/02/2021
22	Ireland	Aileen	74	City-suburb	05/03/2021
23	Ireland	Maureen	76	Rural Town	11/03/2021
24	Ireland	Kaleb	70	City-suburb	15/03/2021
25	Ireland	Frank	64	Rural Area	16/03/2021
26	Ireland	Killian	68	City-suburb	25/03/2021
27	Ireland	Magret	80	Rural Town	26/03/2021
28	Ireland	Audrey	81	Rural Area	01/04/2021
29	Ireland	Thomas	72	City-suburb	21/04/2021

* Couple interview.
